# Impaired functionality of antigen presenting cells in HIV- exposed uninfected infants in the first six months of life

**DOI:** 10.3389/fimmu.2022.960313

**Published:** 2022-08-12

**Authors:** Emilie Jalbert, Tusharkanti Ghosh, Christiana Smith, Fabiana R. Amaral, Marisa M. Mussi-Pinhata, Adriana Weinberg

**Affiliations:** ^1^ Department of Pediatrics, University of Colorado-Denver Anschutz Medical Campus, Aurora, CO, United States; ^2^ Department of Biostatistics and Informatics, Colorado School of Public Health, University of Colorado-Denver Anschutz Medical Campus, Aurora, CO, United States; ^3^ Department of Pediatrics, Ribeirão Preto Medical School, University of São Paulo, Ribeirão Preto, Brazil; ^4^ Department of Medicine and Pathology, University of Colorado-Denver Anschutz Medical Campus, Aurora, CO, United States

**Keywords:** HIV-exposed uninfected (HEU) infants, antigen presenting cell (APC), natural killer cell (NK cell), immune activation, immune regulation, immune responses

## Abstract

HIV-exposed uninfected infants (HEU) have increased morbidity and mortality due to infections in the first 6 months of life that tapers down to 2 years of life. The underlying immunologic defects remain undefined. We investigated antigen-presenting cells (APC) by comparing the phenotype of unstimulated APC, responses to toll-like receptor (TLR) stimulation, and ability to activate natural killer (NK) cells in 24 HEU and 64 HIV-unexposed infants (HUU) at 1-2 days of life (birth) and 28 HEU and 45 HUU at 6 months of life. At birth, unstimulated APC showed higher levels of activation and cytokine production in HEU than HUU and stimulation with TLR agonists revealed lower expression of inflammatory cytokines and activation markers, but similar expression of IL10 regulatory cytokine, in APC from HEU compared to HUU. Differences were still present at 6 months of life. From birth to 6 months, APC underwent extensive phenotypic and functional changes in HUU and minimal changes in HEU. TLR stimulation also generated lower NK cell expression of CD69 and/or IFNγ in HEU compared with HUU at birth and 6 months. *In vitro* experiments showed that NK IFNγ expression depended on APC cytokine secretion in response to TLR stimulation. *Ex vivo* IL10 supplementation decreased APC-mediated NK cell activation measured by IFNγ expression. We conclude that APC maturation was stunted or delayed in the first 6 months of life in HEU compared with HUU. Deficient inflammatory APC responses and/or the imbalance between inflammatory and regulatory responses in HEU may play an important role in their increased susceptibility to severe infections.

## Introduction

More than 1 million HIV-exposed uninfected infants (HEU) are born annually. Compared to HIV-unexposed uninfected infants (HUU), HEU have a 2- to 4-fold higher mortality rate, largely due to infections (1–14). This constitutes a grave public health problem worldwide, particularly in low-and-middle-income countries. Even in resource-rich countries with more advanced health care access, HEU infants have higher hospitalization rates than HUU (12, 15). Mitigation strategies have included breastfeeding and use of cotrimoxazole. The efficacy of these strategies to reduce the morbidity of infections in HEU has not been confirmed (16, 17). There is a great need to identify the immune defects underlying the excess infectious morbidity and mortality in HEU and directly address them.

Much of the excess morbidity and mortality in HEU is due to severe respiratory tract infections caused by respiratory viruses and *S. pneumoniae* (14, 18, 19). HEU generally have lower maternal antibodies against many pathogens compared with HUU (19–21). However, we recently found that antibody titers against respiratory viruses or *S. pneumoniae* in the first day of life did not predict the risk of serious lower respiratory tract infections in HEU (22). Moreover, HEU mount robust antibody responses to vaccines and viral infections suggesting that humoral immunity is conserved and does not contribute to the morbidity and mortality of infections in HEU (20, 23–28).

HEU have T cell, NK, and antigen-presenting cell (APC) defects that may account for the increased infectious morbidity and contribute to decreased T cell responses to vaccines (21, 29–34). We previously showed that NK cell dysfunction in HEU was associated with low IL-12 secretion in PBMC cultures, which we tentatively ascribed to decreased APC functionality (35). Other investigators showed both increased and decreased APC inflammatory responses to a variety of toll-like receptor (TLR) and other nonspecific stimulants in HEU compared with HUU (36–39). Some studies have inferred the APC function based on cytokine levels of bulk PBMC cultures (36, 37). This method is very sensitive but may measure cytokines produced by PBMC other than APC. To devise mitigation strategies of immune dysfunctions in HEU, there is a need to understand the pathophysiology at the cellular level.

The goal of our study was to identify dysfunctional APC responses in HEU by comparing them with HUU. To address this goal, we used high-dimensional flow cytometry to characterize the phenotypic and functional profile of monocyte and dendritic cell (DC) subsets and their interactions with NK and T cells in HEU by comparison with HUU at birth and 6 months of age, coinciding with the time of highest HEU vulnerability to infections.

## Participants and methods

### Participants

The study used viably cryopreserved PBMC collected from 42 HEU and 69 HUU at birth and from 39 HEU and 64 HUU at 6 months of age from 3 cohort studies conducted in South America: the *Eunice Kennedy Shriver* National Institute of Child Health and Human Development (NICHD) International Site Development Initiative (NISDI) Perinatal Study (Perinatal), the NISDI Longitudinal Study in Latin American Countries (LILAC) and Correlações Imunes de Infecções Respiratórias Agudas (CIRAI) Protocol. The demographic and clinical characteristics of these cohorts have been previously published and are also displayed in **Table S1** in the supplemental information (19). Each infant contributed data at a single time point. The subset of infants who contributed samples to this study and the selection criteria were previously described (19). Briefly, we enrolled term infants from singleton pregnancies with birth weight ≥2500 g and absence of congenital defects. Mothers with HIV were treated during pregnancy with zidovudine or combination therapy as per local standards at the time the studies were conducted. HEU were exclusively formula-fed. In an effort to minimize the potential bias introduced by breastfeeding, HUU were enrolled only if they received no more than 50% breast milk, an arbitrarily established threshold. The studies were approved by local and national review boards and the parents/custodians of the participants provided written informed consent.

### Samples

Peripheral blood was collected at 1 day and 6 months of age by venipuncture. PBMC were processed per a cross network consensus protocol (https://www.hanc.info/resources/sops-guidelines-resources/laboratory/cross-network-pbmc-processing-sop.html). Cells were cryopreserved in heat-inactivated FBS+10% dimethylsulfoxide (DMSO), stored and shipped in liquid nitrogen tanks.

### Flow cytometry

Cryopreserved PBMC were thawed in RPMI (Corning) supplemented with L-glutamine (2mM, Gemini), heat-inactivated fetal bovine serum (FBS; 10%, Gemini) and benzonase (50 units/mL, Millipore). Cells were counted with a Guava EasyCyte instrument (Luminex) and Guava ViaCount reagent. Cells were used in one or more panels based on the viable recovery. The PBMC viability and viable recovery were lower in HEU compared with HUU (viability of 40%-83% in HEU vs. 53%-98% in HUU; viable recovery of 0.4*10^6^-3.2*10^6^ in HEU vs. 1.4*10^6^-11.5*10^6^ in HUU), such that each HEU generally contributed to a single panel per time point and HUU to multiple panels. The minimum number of cells required for including a sample in a panel was set *a priori* at 500,000 viable PBMC per stimulated or unstimulated condition. After counting, cells were stimulated for 8 hours or for 24 hours in complete RPMI + 10% FBS [cRPMI; L-glutamine (2mM), Hepes (20mM, Corning), Penicillin : Streptomycin (100U/ml penicillin G and 100µg/ml Streptomycin, Gemini)] with or without a TLR agonist cocktail (TLR B) containing heat-killed *Streptococcus pneumoniae* (TLR2 agonist) 10^6^ cells/ml (*In vivo*gen), lipopolysaccharide (LPS) from E. coli O55:B5 (TLR4 agonist) 1ug/ml (SIGMA), R848 (Resiquimod; TLR7/8 agonist) 1ug/ml (Mabtech), CpG oligonucleotides (ODNs; TLR9 agonist) 1uM/ml (*In vivo*gen). Brefeldin-A and Monensin (5µg/ml each, SIGMA) were added for the last 4h of culture. In all assays, cells were washed with PBS and stained with Zombie Yellow Fixable Viability dye (Biolegend). Cells were then washed with PBS+1%BSA, treated with Human TruStain FcX™ (Fc Receptor Blocking Solution) and washed again. For the 8h stimulation, cells were surface-stained with CD14 Alexa Fluor 488, CD123 PerCP-Cy5.5, CD1c Alexa Fluor 700, CD3 PE-Cy7, CD56 PE-Cy7, CD19 PE-Cy7, CD20 PE-Cy7, HLA-DR APC-H7, CD83 BUV737 (BD Biosciences), brilliant stain buffer plus and True-stain monocyte blocker. Cells were washed and treated with BD FACS lysing solution and with BD FACS permeabilizing solution 2 and stained with TNFα BUV395, IL-6 APC, IL-1b PE, IL-10 BV605, IL-12 BV421 (BD Biosciences) and brilliant stain buffer plus. For the 24h stimulation, cells were surface-stained with CD14 Alexa Fluor 488, CD123 PerCP-Cy5.5, CD1c Alexa Fluor 700, PD-L1 BV605 (Biolegend), CD16 PE-CF594, CD19 PE-Cy7, CD20 PE-Cy7, HLA-DR APC-H7, CD40 APC, CD3 BUV737, CD56 BV650 (BD Biosciences), CD69 PE-Cy5 (Biolegend) with brilliant stain buffer plus and True-stain monocyte blocker. Cells were washed and treated with BD FACS lysing solution and with BD FACS permeabilizing solution 2 and stained with IL-8 BV421, IFNγ BUV395 (BD Biosciences), IL-27 PE (eBiosciences) and brilliant stain buffer plus. In all assays, cells were washed and fixed with PBS+1% paraformaldehyde before acquisition on the cytometer (BD LSRII). The testing laboratory is CLIA-certified, and all assays are optimized and validated as per CLIA regulations (https://www.fda.gov/medical-devices/ivd-regulatory-assistance/clinical-laboratory-improvement-amendments-clia). To minimize biases introduced by inter-assay variability, all study samples were tested in 6 batches, which included PBMC from birth and 6-month HEU and HUU. PBMC from a study-dedicated leukopack control were included in each of the 6 runs and used to verify inter-assay reproducibility. Runs were considered valid if the leukopack results were within mean ± 2 standard deviations, established by testing 10 aliquots of the leukopack in multiple replicates in the same run and on different days. The gating strategy is shown in **Figure S1**.

### Mechanistic experiments

#### Blockade of cytokine export

Cryopreserved PBMC from 3 HUU at birth were thawed and counted as described above. Cells were stimulated for 24h in complete RPMI+10% FBS with TLR cocktail in duplicate. BFA (5ug/mL) was added to one replicate at the beginning of the stimulation, or for the last 4 hours of culture to the second replicate. Cells were washed with PBS, stained with Zombie Yellow Fixable Viability dye and surface stained with HLA-DR Alexa Fluor 488, PD-L1 BV421, CD19 PerCP-Cy5.5, CD69 APC-Cy7 (Biolegend), CD56 PE, CD16 PE-CF594, CD14 PerCP-Cy5.5, CD20 PerCP-Cy5.5 and CD3 Alexa Fluor700 (BD Biosciences). Cells were washed and treated with BD lysing solution and BD permeabilization buffer 2 (BD Biosciences) then stained for IL-8 PE-Cy7 (Biolegend) and IFNγ APC (eBiosciences). Cells were washed and fixed with PBS+1% paraformaldehyde before acquisition on the cytometer (Beckman Coulter Gallios).

#### IL-10 supplementation and neutralization

Cryopreserved PBMC (5 HUU birth and 5 HUU 6-month samples) were thawed and counted as described above. Cells were stimulated for 24h in complete RPMI+10% FBS with TLR cocktail alone or in combination with rhIL-10 (20ng/mL, R&D Systems) or anti-hIL-10 (500ng/mL, R&D Systems). BFA (5ug/mL) was added for the last 4 hours of culture. Cells were washed with PBS and stained with Zombie Yellow Fixable Viability, followed by surface staining with HLA-DR Alexa Fluor 488, CD19 PerCP-Cy5.5, CD16 APC, CD56 APC-Cy7 (Biolegend), Gamma delta TCR PE and CD3 Alexa Fluor 700 (BD Biosciences). Cells were washed and treated with eBioscience Foxp3/Transcription Factor Staining Buffer Set (ThermoFisher) then stained for IL-10 PE-Dazzle 594, IFNγ BV421 (Biolegend) and FoxP3 PE-Cy7 (eBiosciences). Cells were washed and fixed with PBS+1% paraformaldehyde before acquisition on the cytometer (Beckman Coulter Gallios).

### Statistical analysis

To evaluate phenotypic differences by HIV exposure status, HEU versus HUU, at a given timepoint, we used logistic regression to model each participant’s cell subset frequency by exposure group in unstimulated conditions. We defined significant differences between exposure groups at a given timepoint as a non-zero HEU/HUU logistic regression coefficient with multiple testing adjustment using a 5% Benjamini-Hochberg False Discovery Rate (FDR). For functional differences, we subtracted cell subset frequencies in unstimulated from TLR B-stimulated conditions and repeated the same process. Because of the systematic lower viable recovery of HEU than HUU PBMC, we identified cell subset comparisons that were significantly associated with cell viability by using the viability as a covariate in the regression model. To avoid potential biases introduced by the viable recovery, we excluded from the report subset comparisons that were significantly associated with the cell viability. For longitudinal differences, we used the same analytical process to compare birth and 6 months results. Since viability and viable recovery did not differ between birth and 6 months within each group of HEU and HUU, no exclusions were made based on the relationship with cell viability. All the analyses above were performed in R (version 4.1.3). The results of the mechanistic experiments were analyzed using Prism software (Graphpad).

## Results

### Phenotypic characteristics of APC in HEU and HUU

After exclusion of samples with viable recovery <500,000 PBMC, we analyzed activation and cytokine expression in stimulated and unstimulated PBMC from 24 HEU and 64 HUU at birth and 28 HEU and 45 HUU at 6 months of age.

**Figure 1:**
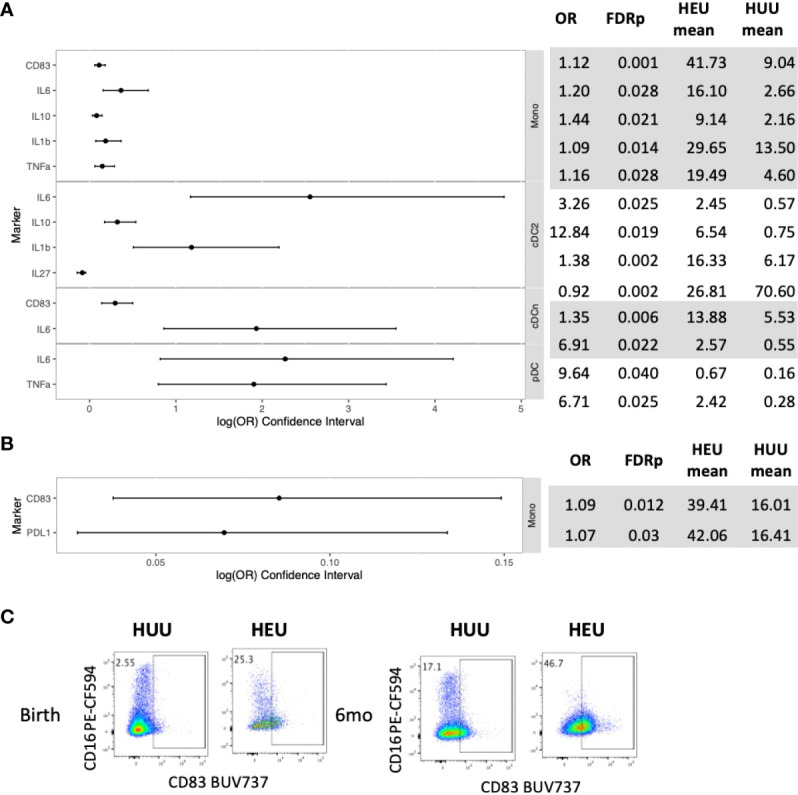
Differential phenotypic characteristics of APC in HEU and HUU at birth and 6 months of life. Data were derived from 24 HEU at birth, 28 HEU at 6 months, 64 HUU at birth and 45 HUU at 6 months. **(A)** Forest plot and summary statistics of significant differences in HEU and HUU at birth (OR, odds ratio; FDRp, p values after FDR correction; mean, mean proportion of cells expressing the marker of interest in unstimulated conditions). **(B)** Forest plot and summary statistics of significant differences in HEU and HUU at 6 months of life. **(C)** Representative scatter plots of CD83+ Mono (Lin-HLADR+CD14+) illustrating phenotypic (unstimulated conditions) differences between HEU and HUU at birth and 6 months of life in unstimulated conditions.

In the phenotypic analysis of unstimulated PBMC at birth, compared with HUU, HEU had 12 (48%) out of 25 evaluable cell subsets with significantly higher activation or cytokine expression and one subset with lower activation (**Figures 1A, C**; **Table S1**). The activated cell subsets higher in HEU than HUU included monocytes (Mono; Lin-HLADR+CD14+), conventional dendritic cells type 2 (cDC2; Lin-HLADR+CD14-CD123-CD1c+), conventional dendritic cells other than type 2 (cDCn; Lin-HLADR+CD14-CD123- CD1c-) and plasmacytoid dendritic cells (pDC; Lin-HLADR+CD14-CD123+CD11c-) expressing CD83, IL1β, IL6, IL10 and/or TNFα. At 6 months of life, only 2 out of 34 evaluable subsets (6%), consisting of Mono expressing CD83 and PDL-1, had significantly higher frequencies in HEU compared with HUU (**Figures 1B, C**; **Table S2**).

### Functional characteristics of APC in HEU and HUU

**Figure 2:**
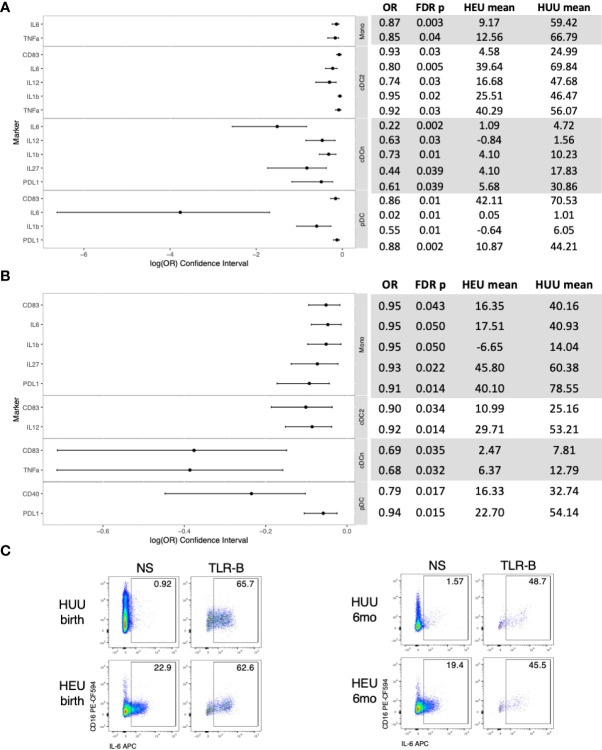
Differential functional characteristics of APC in HEU and HUU at birth and 6 months of life. Data were derived from 24 HEU at birth, 28 HEU at 6 months, 64 HUU at birth and 45 HUU at 6 months. **(A)** Forest plot and summary statistics of significant differences in HEU and HUU at birth. Means indicate proportion of cells expressing the marker of interest in TLR-stimulated conditions (TLR-B) after subtraction of the proportion of cells in unstimulated conditions (NS). **(B)** Forest plot and summary statistics of significant phenotypic differences in HEU and HUU at 6 months of life. **(C)** Representative scatter plots of IL6+ Mono (Lin-HLADR+CD14+) illustrating differences between HEU and HUU at birth and 6 months of life.

At birth, compared with HUU, the TLR agonist *ex vivo* stimulation generated lower activation and inflammatory cytokine production in 16 (70%) out of 23 evaluable cell subsets in HEU and there were no cell subsets with higher frequencies (**Figures 2A, C**; **Table S3**). In contrast, IL10 production, the only regulatory cytokine included in the panel, did not significantly differ between HEU and HUU.

At 6 months of age, compared with HUU, the *ex vivo* stimulation generated lower activation and inflammatory cytokine production in 11 (39%) out of 28 evaluable cell subsets in HEU and there were no cell subsets with higher frequencies (**Figures 2B, C**; **Table S2**). IL10 production did not significantly differ between HEU and HUU.

### Maturation of APC functionality in HEU and HUU

**Figure 3:**
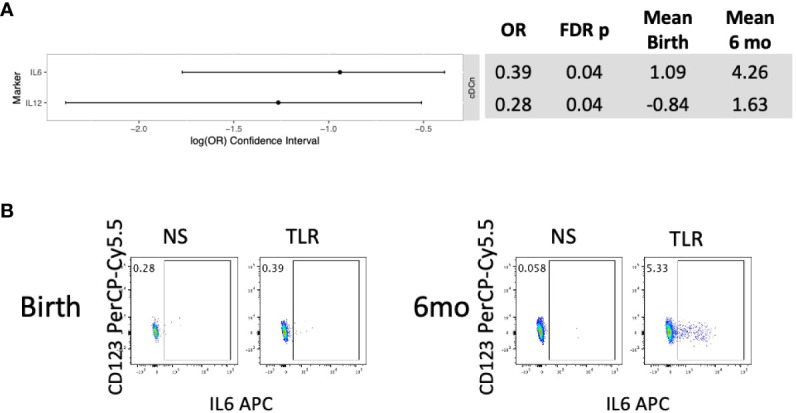
Differential functional characteristics of APC in HEU between birth and 6 months of life. Data were derived from 24 HEU at birth and 28 HEU at 6 months. **(A)** Forest plot and summary statistics of significant differences. **(B)** Representative scatter plots of IL6+ cDCn (Lin-HLADR+CD14-CD123-CD1c-) illustrating differences between HEU and HUU at birth and 6 months of life.

In HEU, unstimulated APC did not show significant changes in phenotypic characteristics between birth and 6 months of life (**Table S4**). In stimulated conditions, HEU showed significant increases in production of IL6 and IL12 in cDCn and no other significant changes (**Figure 3**; **Table S3**).

**Figure 4:**
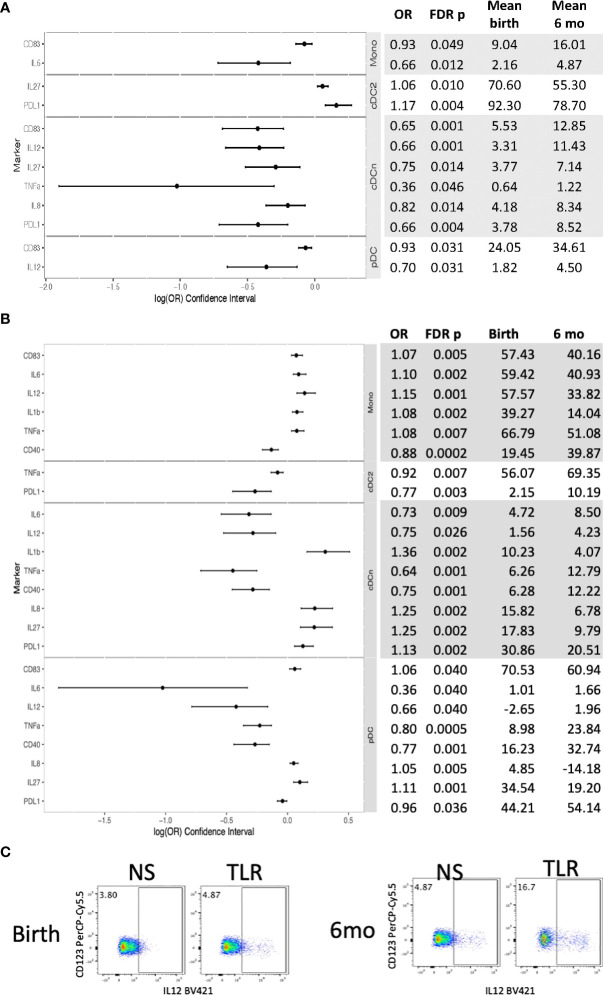
Differential phenotypic and functional characteristics of APC in HUU between birth and 6 months of life. Data were derived from 64 HUU at birth and 45 HUU at 6 months. **(A)** Forest plot and summary statistics of significant phenotypic differences. **(B)** Forest plot and summary statistics of significant functional differences. **(C)** Representative scatter plots of IL12+ cDCn (Lin-HLADR+CD14-CD123-CD1c-) illustrating differences between birth and 6 months of life.

In contrast, in HUU, 12 unstimulated APC subsets significantly changed marker and cytokine expression between birth and 6 months of life (**Figures 4A, C**; **Table S4**). Mono, cDCn and pDC increased expression of CD83, PDL-1, IL6, IL8, IL12 and/or TNFα, while cDC2 decreased expression of PDL-1 and IL27 from birth to 6 months of life. Stimulation generated significant changes in 24 outcome measures (**Figures 4B, C**; **Table S5**). Overall, expression of CD83, IL1β, IL8, and IL27 decreased from birth to 6 months of age; CD40 increased; and IL6, IL12, TNFα, and PDL-1 increased in some APC subsets and decreased in other subsets. Among APC subsets, cDC2 activation and cytokine production increased from birth to 6 months of age and monocyte activation and cytokine production generally decreased.

### NK cell responses to APC stimulation at birth and 6 months of life in HEU and HUU

**Figure 5:**
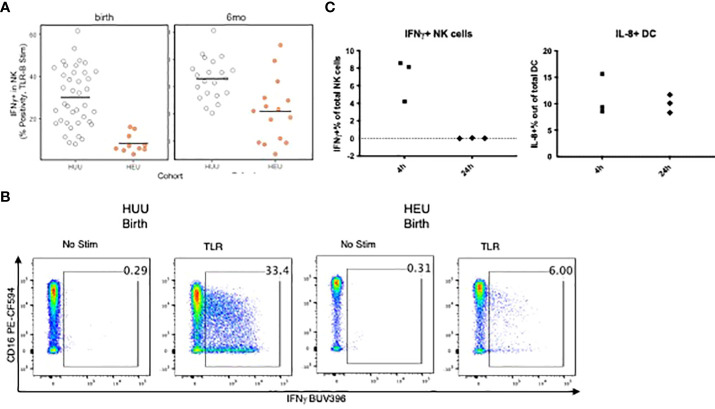
Requirement of APC-secreted factors for NK cell activation in TLR-stimulated PBMC. **(A)**: Scatter plots comparing NK expression of IFNg in HEU and HUU PBMC stimulated with TLR agonists for 24 h Data were derived from 10 HEU at birth, 15 HEU at 6 months, 39 HUU at birth and 21 HUU at 6 months. Dots represent individual participant results. Horizontal lines indicate the medians. **(B)** Representative flow scatter plots for the differences in IFNg+ NK cells between HEU and HUU. **(C)** Scatter plots show the effect of blocking cytokine secretion for the entire 24-h stimulation period compared with blocking for the last 4 h of stimulation on activation of NK cells (left) and DC (right). The dots represent the frequency of activated cells in TLR-stimulated conditions after subtraction of unstimulated control. Blocking cytokine export during the entire 24 h period of stimulation completely abrogated NK cell responses, but not DC responses, indicating that NK responses depended on APC-derived cytokine stimulation, whereas DC were directly stimulated by the TLR agonists.

The ability of APC to stimulate NK and T cells was measured by CD69 and IFNγ expression after 24h of PBMC stimulation with TLR agonists. T cell activation did not significantly differ between groups. However, compared with HUU, NK cells of HEU showed lower CD69 (not depicted) and IFNγ responses at birth and lower IFNγ at 6 months of life (**Figures 5A–C**). To verify that the NK cell activation in PBMC cultures stimulated with the TLR agonists was dependent on APC, we compared NK cell activation in the presence of Brefeldin A added to the PBMC for the entire duration of the stimulation or only for the last 4h. Brefeldin A blocks protein export and precludes cytokine-mediated communications between APC and NK cells. The results of these experiments using PBMC from 3 infants obtained at birth showed that IFNγ expression on NK cells increased in response to the TLR cocktail when BFA was added for the last 4h of stimulation, but not from the beginning of the stimulation (**Figure 5C**). In contrast, DC intracellular IL-8 production was similar in both experimental conditions.

### Significance of imbalance between IL-10 and inflammatory cytokine production in APC

**Figure 6:**
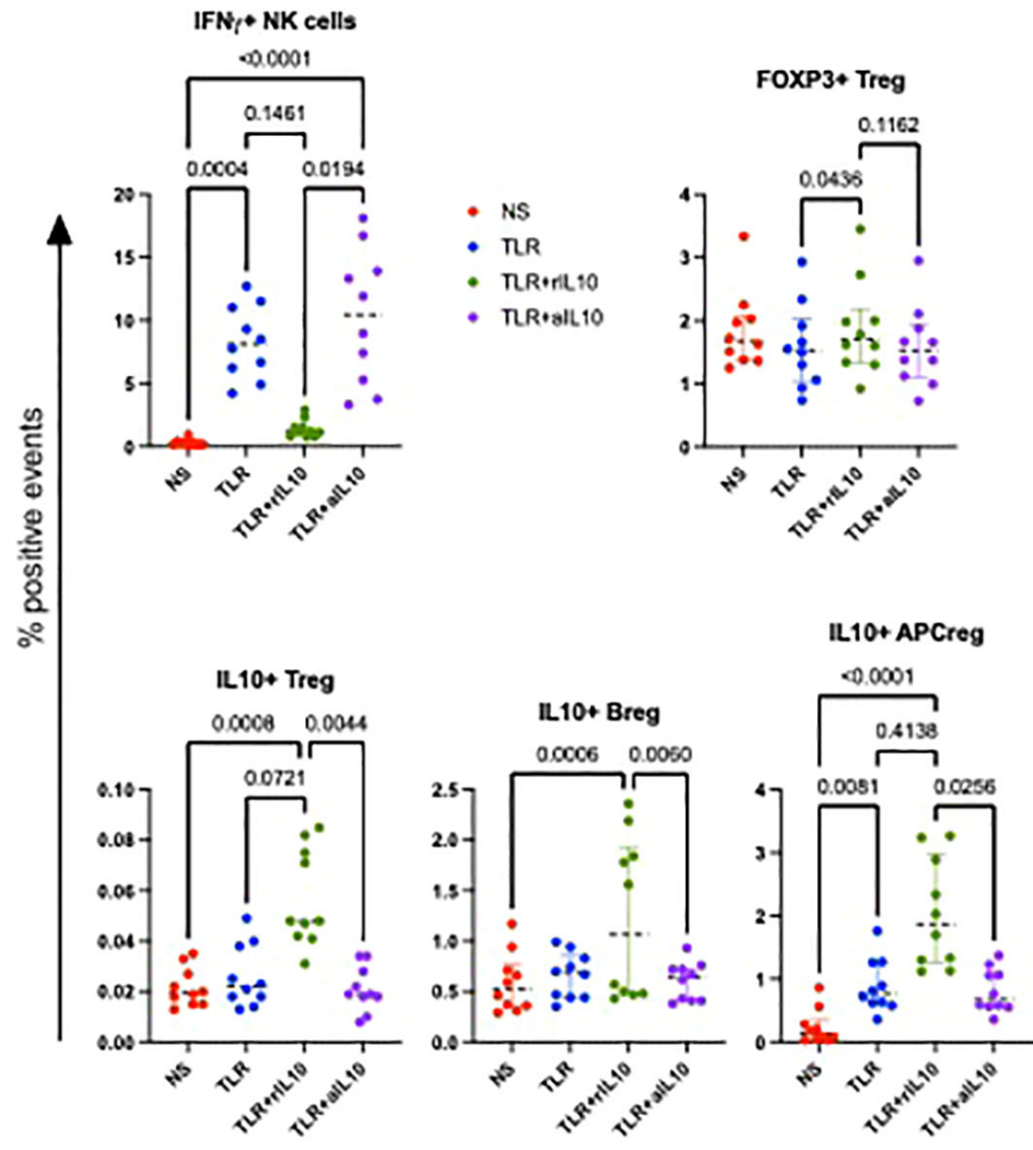
Effect of rhIL10 and anti-hIL10 treatment of TLR-stimulated PBMC cultures. Data were derived using PBMC from 5 HUU at birth and 5 HUU at 6 months of life. Scatter plots show individual sample results, medians, and select p values calculated by Friedman’s test for nonparametric repeated measures using Prism software (Graphpad).

We hypothesized that the imbalance between IL-10 and inflammatory cytokine production in APC contributed to decreased NK activation and effector functions in HEU compared with HUU. To test this hypothesis, we added pre-optimized concentrations of rhIL-10 or neutralizing anti-IL-10 antibodies to PBMC cultures stimulated with TLR agonists. Using PBMC from 5 HUU at birth and 5 HUU at 6 months, we found that rhIL-10 supplementation expanded CD3+ regulatory T cells (Treg) expressing IL-10 or FOXP3, regulatory IL-10-secreting regulatory B cells (Breg), and IL-10 secreting regulatory APC (**Figure 6**). In addition, rhIL-10 treatment decreased NK cell IFNγ production in TLR-stimulated PBMC. The addition of anti-IL-10 neutralizing antibodies to TLR-stimulated PBMC abrogated the effect of IL-10 supplementation on IFNγ expression in NK cells and on the generation of Treg, Breg and regulatory APC, authenticating the functional effect of IL-10 supplementation.

## Discussion

This comprehensive analysis of the APC phenotypic and functional characteristics in HEU and HUU showed significant differences between the two groups at birth that tended to decrease without disappearing over the first 6 months of life. The convergence of HEU and HUU APC functionality over time was consistent with the time course of their increased susceptibility to infections, which gradually decreases over the first 2 years of life (40). The medical literature includes conflicting results on the differences in APC functional responses between HEU and HUU (37–39, 41). In agreement with our results, Chougnet et al. showed decreased production of IL-12 in stimulated CBMC in HEU compared with HUU (37). However, other investigators noted increased inflammatory responses to TLR stimulation in HEU compared with HUU (38, 39). All studies invariably found higher baseline activation in APC from HEU compared with HUU. Thus, although the baseline activation contributed in some cases to the net decreased response to *ex vivo* stimulation in our study, this was probably the case in previous studies, too. There were differences between our study and previous studies with respect to outcome measures, stimulants, and the country of origin of the study participants, all of which might have contributed to the discrepancies noted above.

Of note, the high baseline activation of specific cell subsets in HEU, including IL12+ cDCn and IL1β+ pDC at birth, and IL1β+ Mono at 6 months of age, was associated with a negative net result after subtraction of unstimulated from the *ex vivo*-stimulated cell subsets. This observation suggests that these cell subsets reached a level of activation under steady state conditions that precluded a response to additional *ex vivo* stimulation. The corollary of this observation is that people with high inflammatory underlying health conditions may not be able to mount optimal responses when exposed to new pathogens. This hypothesis needs additional investigation.

The increased activation of APC in HEU at birth may originate from the fetal exposure to a highly inflammatory maternal environment. Women with HIV on effective ARV, including pregnant women, have higher levels of inflammation compared with women without HIV (42–45). Although the means of communication between maternal and fetal immune systems are not well understood, there is abundant evidence that maternal characteristics, including HIV and other chronic infections, type 1 diabetes, and obesity, imprint the fetal immune system (46–52). Unfortunately, effective antiretroviral therapy has been insufficient to abate the highly inflammatory environment characteristic of HIV Infection (43, 53–56). Additional interventions may be necessary to avoid fetal immune activation in the context of maternal HIV infection.

The differences in APC phenotype and function between HEU and HUU at 6 months of age were accompanied by differences in maturation. While HUU showed significant changes in APC phenotypic and functional characteristics from birth to 6 months, HUU did not, suggesting that the APC maturation process may be stunted or delayed in HEU.

Our *ex vivo* experiments suggested that decreased APC functionality might play a critical role in the decreased NK cell activation and/or IFNγ production in HEU compared with HUU. Although NK cells express TLR, TLR agonists have been shown to exclusively amplify responses triggered by engagement of other NK activating receptors (57–59). Of note, the results of our experiments did not rule out additional intrinsic NK cell dysfunctions.

An important observation was the imbalance between the APC production of inflammatory cytokines and IL-10 in response to TLR stimulation in HEU compared to HUU. The potential role of this imbalance in dampening immune cell functionality was supported by *ex vivo* experiments in which addition of rhIL-10 to TLR-stimulated PBMC from HUU increased the frequency of Treg and Breg and decreased NK functional responses, which were at least partially restored with anti-IL-10 neutralizing antibodies. Since we did not have any remaining samples from HEU for mechanistic experiments, we could not study the effect of anti-IL10 antibodies on NK functionality in HEU. However, in a previous study, we showed lower cytotoxicity of NK cells in HEU compared with HUU, which could be corrected with supplementation of rhIL-12 (35).

This study was limited by the low viable recovery of PBMC in HEU, which prevented us from running multiple panels on the same sample and translated into a relatively small number of data points per panel in HEU. The low number of HEU samples per assay may have led to underestimation of differences between HEU and HUU at 6 months of age and between birth and 6 months in HEU. Because the viability of PBMC was also lower in HEU compared to HUU, we reported results in the comparison of HEU with HUU only for the analytes that were not significantly associated with viability, which also decreased the number of parameters reported. Strengths of this study include the similar geographic location, race, ethnicity, term births and maternal substance use during pregnancy in cases and controls (19). Thus, differences in APC functionality at birth were likely exclusively due to the maternal HIV infection status. However, lack of breastfeeding and other unknown factors might have affected the development of APC functionality in HEU and HUU and contributed to the differences that we observed.

In conclusion, APC in HEU compared with HUU displayed higher levels of baseline activation, but decreased expression of activation markers and inflammatory cytokine production in response to TLR stimulation, that extended from birth up to at least 6 months of life. The impaired functionality of APC also decreased their ability to stimulate NK responses. Collectively, the deficit in the innate immune response may contribute to the increased susceptibility to severe infections in HEU.

## NICHD International Site Development Initiative Perinatal/LILAC Protocol

Principal investigators, *co-principal investigators*, study coordinators, data management center representatives, and NICHD staff include: **Argentina: *Buenos Aires*:** Marcelo H. Losso, Irene Foradori, Alejandro Hakim, Silvina Ivalo, Erica Stankievich (Hospital General de Agudos José María Ramos Mejía); **Brazil: *Belo Horizonte:*** Jorge A. Pinto, *Victor H. Melo*, Flávia F. Faleiro, Fabiana Kakehasi, Beatriz M. Andrade (Universidade Federal de Minas Gerais); ***Caxias do Sul***: Rosa Dea Sperhacke, *Nicole Golin*, Sílvia Mariani Costamilan (Universidade de Caxias do Sul/Serviço Municipal de Infectologia); ***Nova Iguacu:*** Jose Pilotto, *Luis Eduardo Fernandes, Ivete Gomes, Luis Felipe Moreira*, Gisely Falco (Hospital Geral Nova de Iguacu – HIV Family Care Clinic); ***Porto Alegre:*** Rosa Dea Sperhacke, *Breno Riegel Santos*, Rita de Cassia Alves Lira (Universidade de Caxias do Sul/Hospital Conceição); Rosa Dea Sperhacke, *Mario Ferreira Peixoto*, Elizabete Teles (Universidade de Caxias do Sul/Hospital Fêmina); Rosa Dea Sperhacke, *Marcelo Goldani*, Carmem Lúcia Oliveira da Silva, Margery Bohrer Zanetello (Universidade de Caxias do Sul/Hospital de Clínicas de Porto Alegre); Regis Kreitchmann, *Fabrizio Motta*, *Luis Carlos Ribeiro*, *Marcelo Comerlato Scotta*, Debora Fernandes Coelho (Irmandade da Santa Casa de Misericordia de Porto Alegre); ***Ribeirão Preto*:** Marisa M. Mussi-Pinhata, *Maria Célia Cervi, Geraldo Duarte*, Fabiana Rezende Amaral, Adriana A. Tiraboschi Bárbaro, Conrado Milani Coutinho, Márcia L. Isaac, Anderson Sanches de Melo, Bento V. Moura Negrini, Fernanda Tomé Sturzbecher (Hospital das Clínicas da Faculdade de Medicina de Ribeirão Preto da Universidade de São Paulo); ***Rio de Janeiro:*** Ricardo Hugo S. Oliveira, *Elizabeth S. Machado*, Maria C. Chermont Sapia (Instituto de Puericultura e Pediatria Martagão Gesteira**);** Esau Custodio Joao, *Maria Leticia Cruz*, Maria Isabel Gouvêa, Leon Claude Sidi, Mariza Curto Saavedra, Clarisse Bressan, Fernanda Cavalcanti A. Jundi (Hospital dos Servidores do Estado); ***São Paulo:*** Regina Celia de Menezes Succi, Prescilla Chow, Daisy Maria Machado (Escola Paulista de Medicina- Universidade Federal de São Paulo); Marinella Della Negra, *Yu Ching Lian*, *Wladimir Queiroz* (Instituto de Infectologia Emilio Ribas); **Mexico: *Mexico City:*** Noris Pavía-Ruz, Karla Ojeda-Diezbarroso*, Dulce Morales-Pérez* (Hospital Infantil de México Federico Gómez); **Peru: *Lima:*** Jorge O. Alarcón Villaverde (Instituto de Medicina Tropical “Daniel Alcides Carrión”- Sección de Epidemiologia, UNMSM), *María Castillo Díaz, Carlos Velásquez Vásquez* (Instituto Nacional de Salud del Niño), Mary Felissa Reyes Vega, César Gutiérrez Villafuerte (Instituto de Medicina Tropical “Daniel Alcides Carrión” - Sección de Epidemiologia, UNMSM); **Data Management and Statistical Center:** Yolanda Bertucci, Rachel Cohen, Laura Freimanis-Hance, René Gonin, D. Robert Harris, Roslyn Hennessey, James Korelitz, Margot Krauss, Sue Li, Karen Megazzini, Orlando Ortega, Sharon Sothern de Sanchez, Sonia K. Stoszek, Qilu Yu (Westat, Rockville, MD, USA); **NICHD:** Rohan Hazra, George K. Siberry, Lynne M. Mofenson (*Eunice Kennedy Shriver* National Institute of Child Health and Human Development, Bethesda, Maryland).

**Correlações Imunes De Infecções Respiratórias Agudas (CIRAI) Protocol** includes the following research teams: **Ribeirao Preto, SP, Brazil**: Maria M. Mussi-Pinhata, MD, Volia C. Almeida, MD, Fabiana R. Amaral, Tatiana C.B. Mota, Cleonice B.S. Souza, Mariani B. Ferracini, Audery K.A. Silva, Vanilda R.B. Amaral; **Anschutz Medical Center, University of Colorado Denver, CO, USA**: Adriana Weinberg.

## Data availability statement

The raw data supporting the conclusions of this article will be made available by the authors, without undue reservation.

## Ethics statement

Ethical review and approval was not required for the study on human participants in accordance with the local legislation and institutional requirements. Written informed consent to participate in this study was provided by the participants’ legal guardian/next of kin.

## Author contributions

EJ performed laboratory assays and contributed to data analysis and manuscript preparation; TG performed statistical analyses and contributed to manuscript preparation; CS contributed to study design and manuscript preparation; FA organized recruitment and specimen collection from study participants; MM-P oversaw recruitment, specimen collection and processing; AW designed the study, analyzed the data, and wrote the manuscript. All authors reviewed and approved the manuscript before submission.

## Funding

Contract HHSN275201300003C NICHD (AW) and U01 AI131360-01 NIAID (AW).

## Acknowledgments

The authors thank Cuining Liu for statistical advice and the participants and personnel at the NISDI Perinatal, LILAC and CIRAI research sites.

## Conflict of interest

The authors declare that the research was conducted in the absence of any commercial or financial relationships that could be construed as a potential conflict of interest.

## Publisher’s note

All claims expressed in this article are solely those of the authors and do not necessarily represent those of their affiliated organizations, or those of the publisher, the editors and the reviewers. Any product that may be evaluated in this article, or claim that may be made by its manufacturer, is not guaranteed or endorsed by the publisher.
