# Supplementary material for: Impaired functionality of antigen presenting cells in HIV- exposed uninfected infants in the first six months of life
Source: Front Immunol. 2022 Aug 12;13:960313. doi: 10.3389/fimmu.2022.960313 (PMC9411519; doi:10.3389/fimmu.2022.960313)
Supplement: Supplementary file 1 [file DataSheet_1.docx]

**Table S1. Demographic characteristics of mothers and infants according to infant HIV exposure category**

| **Variable** | **Infant HIV exposure category** | | **p-value*** |
| --- | --- | --- | --- |
|  | **HEU**  (N = 247) | **HUU**  (N=88) |  |
| ***Maternal Characteristics*** | | | |
| Mean (SD^&^) maternal age at enrollment | 26.9 (5.5) | 25.7 (5.8) | 0.08 |
| Substance use during pregnancy: n (%)  Alcohol  Tobacco  Marijuana  Crack or cocaine  Other recreational drug use | 49 (20.0)  58 (23.8)  9 (3.8)  8 (3.4)  37 (100) | 9 (10.2)  12 (13.6)  0 (0.0)  0 (0.0)  88 (100) | 0.05  0.05  0.12  0.11  - |
| Mean (SD) years of completed education | 7.4 (3.2) | 9.3 (2.4) | <.0001 |
| Mean (SD) number of prior pregnancies | 2.3 (2.1) | 1.1 (1.2) | <.0001 |
| Mean (SD) parity | 1.7 (1.6) | 0.9 (1.1) | <.0001 |
| Mean (SD) number of people living in the household | 4.2 (2.5) | 4.5 (1.7) | 0.11 |
| Mean (SD) CD4 count (cells/mm^3^) at hospital discharge | 558.7 (270.7) | - | - |
| Mean (SD) CD4% at hospital discharge | 31.7 (10.5) | - | - |
| Mean (SD) viral load (log_10_ copies/mL) at hospital discharge | 2.62 (0.81) | - | - |
| Viral load <400 copies/mL at hospital discharge: n (%) | 80 (48.2) | - | - |
| ARV use during pregnancy: n (%)  ZDV monotherapy  Combination ARV regimen  Unknown | 127 (51.4)  119 (48.2)  1 (0.4) | -  -  - | -  -  - |
| ***Infant Characteristics*** | | | |
| Mean (SD) gestational age at birth (weeks) | 38.7 (1.1) | 39.3 (1.4) | 0.001 |
| Female gender: n (%) | 100 (40.5) | 43 (48.9) | 0.21 |
| Mean (SD) birth weight (g) | 3159.2 (378.7) | 3382.2 (390.9) | <0.0001 |
| Small for gestational age (<10^th^ percentile): n (%) | 8 (3.2) | 0 (0.0) | 0.12 |
| Large for gestational age (>90^th^ percentile): n (%) | 27 (10.9) | 22 (25.0) | 0.003 |
| Not breastfeeding: n (%) | 247 (100) | 1 (1.1) | <0.0001 |

^&^SD = standard deviation

* All p-values were calculated by two-sample t-test or chi-square/Fischer’s exact test, where appropriate.

**Note**: maternal race was not analyzed due to the inability to establish it in the highly mixed Brazilian population.

**Figure S1. Gating Strategy**

**Table S2. Comparative phenotypic characteristics of HEU and HUU**

| **Age** | **Cell subset** | **Marker** | **OR** | **FDR p** | **HEU_mean** | **HUU_mean** |
| --- | --- | --- | --- | --- | --- | --- |
| **Birth** | cDC2 | CD83 | 0.96 | 0.148 | 54.98 | 63.47 |
|  |  | IL1b | 3.26 | 0.025 | 2.45 | 0.57 |
|  |  | IL6 | 12.84 | 0.019 | 6.54 | 0.75 |
|  |  | IL10 | 1.38 | 0.002 | 16.33 | 6.17 |
|  |  | IL12 | 0.96 | 0.089 | 31.09 | 44.47 |
|  |  | IL27 | 0.92 | 0.002 | 26.81 | 70.60 |
|  |  | TNFa | 1.11 | 0.237 | 4.79 | 3.51 |
|  | cDCn | CD83 | 1.35 | 0.006 | 13.88 | 5.53 |
|  |  | IL1b | 8.95 | 0.569 | 0.28 | 0.19 |
|  |  | IL6 | 6.91 | 0.022 | 2.57 | 0.55 |
|  |  | IL10 | 0.99 | 1.000 | 6.50 | 6.57 |
|  |  | IL12 | 1.00 | 1.000 | 3.31 | 3.31 |
|  |  | TNFa | 2.19 | 0.569 | 0.91 | 0.64 |
|  | Mono | CD83 | 1.12 | 0.001 | 41.73 | 9.04 |
|  |  | IL1b | 1.20 | 0.028 | 16.10 | 2.66 |
|  |  | IL6 | 1.44 | 0.021 | 9.14 | 2.16 |
|  |  | IL10 | 1.09 | 0.014 | 29.65 | 13.50 |
|  |  | IL12 | 1.00 | 0.902 | 57.91 | 56.99 |
|  |  | TNFa | 1.16 | 0.028 | 19.49 | 4.60 |
|  | pDC | CD83 | 1.07 | 0.096 | 32.80 | 24.05 |
|  |  | IL1b | 1.61 | 0.537 | 0.46 | 0.19 |
|  |  | IL6 | 9.64 | 0.040 | 0.67 | 0.16 |
|  |  | IL10 | 0.94 | 0.537 | 5.04 | 8.28 |
|  |  | IL12 | 1.23 | 0.255 | 5.73 | 1.82 |
|  |  | TNFa | 6.71 | 0.025 | 2.42 | 0.28 |
| **6 months** | cDC2 | CD40 | 1.02 | 0.73 | 25.64 | 21.06 |
|  |  | CD83 | 0.99 | 1.000 | 57.16 | 59.76 |
|  |  | IL10 | 1.01 | 1.000 | 8.77 | 8.32 |
|  |  | IL12 | 1.00 | 1.000 | 47.46 | 45.99 |
|  |  | IL1b | 2.88 | 0.116 | 1.85 | 0.58 |
|  |  | IL27 | 0.97 | 0.40 | 42.38 | 55.30 |
|  |  | IL6 | 2.92 | 0.066 | 2.90 | 1.04 |
|  |  | TNFa | 0.87 | 1.000 | 3.36 | 4.11 |
|  | cDCn | CD40 | 0.84 | 0.72 | 1.44 | 2.09 |
|  |  | CD83 | 0.98 | 1.000 | 12.16 | 12.85 |
|  |  | IL10 | 0.98 | 1.000 | 5.35 | 5.56 |
|  |  | IL12 | 0.93 | 0.876 | 8.35 | 11.43 |
|  |  | IL1b | 9.82 | 0.418 | 0.46 | 0.26 |
|  |  | IL6 | 2.76 | 0.876 | 1.09 | 0.76 |
|  |  | TNFa | 0.92 | 1.000 | 1.15 | 1.22 |
|  | Mono | CD40 | 1.67 | 0.13 | 1.80 | 1.04 |
|  |  | CD83 | 1.09 | 0.012 | 39.41 | 16.01 |
|  |  | IL10 | 1.04 | 0.267 | 24.43 | 17.28 |
|  |  | IL12 | 1.05 | 0.090 | 80.68 | 61.63 |
|  |  | IL1b | 1.25 | 0.099 | 24.50 | 2.55 |
|  |  | IL27 | 3.05 | 0.07 | 8.13 | 0.38 |
|  |  | IL6 | 0.89 | 0.267 | 3.41 | 4.87 |
|  |  | IL8 | 1.04 | 0.13 | 77.08 | 62.14 |
|  |  | PDL1 | 1.07 | 0.03 | 42.06 | 16.41 |
|  |  | TNFa | 1.13 | 0.062 | 27.46 | 6.57 |
|  | pDC | CD40 | 1.11 | 0.48 | 5.51 | 3.71 |
|  |  | IL10 | 1.00 | 1.000 | 10.59 | 10.62 |
|  |  | IL12 | 1.12 | 0.863 | 6.91 | 4.50 |
|  |  | IL1b | 3.66 | 0.863 | 0.39 | 0.18 |
|  |  | IL27 | 0.99 | 0.79 | 15.12 | 16.13 |
|  |  | IL6 | 1.63 | 1.000 | 0.57 | 0.18 |
|  |  | IL8 | 1.03 | 0.34 | 45.92 | 34.06 |
|  |  | PDL1 | 1.03 | 0.48 | 35.99 | 28.19 |
|  |  | TNFa | 0.98 | 1.000 | 0.53 | 0.55 |

Cell subsets with no significant associations with PBMC viability.

**Table S3. Comparative functional characteristics of HEU and HUU**

| **Age** | **Cell subset** | **Marker** | **OR** | **FDR p** | **HEU_mean** | **HUU_mean** |
| --- | --- | --- | --- | --- | --- | --- |
| **Birth** | cDC2 | CD83 | 0.93 | 0.035 | 4.58 | 24.99 |
|  |  | IL10 | 1.00 | 0.984 | 0.72 | 0.69 |
|  |  | IL12 | 0.74 | 0.035 | 16.68 | 47.68 |
|  |  | IL1b | 0.95 | 0.023 | 25.51 | 46.47 |
|  |  | IL6 | 0.80 | 0.005 | 39.64 | 69.84 |
|  |  | TNFa | 0.92 | 0.035 | 40.29 | 56.07 |
|  | cDCn | CD83 | 0.86 | 0.258 | 3.32 | 6.27 |
|  |  | IL10 | 0.75 | 0.449 | -0.12 | 0.07 |
|  |  | IL12 | 0.63 | 0.026 | -0.84 | 1.56 |
|  |  | IL1b | 0.73 | 0.005 | 4.10 | 10.23 |
|  |  | IL27 | 0.44 | 0.039 | 4.10 | 17.83 |
|  |  | IL6 | 0.22 | 0.002 | 1.09 | 4.72 |
|  |  | PDL1 | 0.61 | 0.039 | 5.68 | 30.86 |
|  |  | TNFa | 0.86 | 0.440 | 4.66 | 6.26 |
|  | Mono | CD83 | 0.00 | 1.000 | -5.71 | 57.43 |
|  |  | IL10 | 0.97 | 1.000 | -1.20 | -0.55 |
|  |  | IL12 | 0.75 | 0.351 | -0.33 | 57.57 |
|  |  | IL1β | 0.54 | 0.281 | -11.69 | 39.27 |
|  |  | IL6 | 0.87 | 0.003 | 9.17 | 59.42 |
|  |  | TNFα | 0.85 | 0.040 | 12.56 | 66.79 |
|  | pDC | CD83 | 0.86 | 0.011 | 42.11 | 70.53 |
|  |  | IL10 | 0.29 | 0.257 | -0.30 | -0.01 |
|  |  | IL12 | 0.99 | 1.000 | -2.94 | -2.65 |
|  |  | IL1b | 0.55 | 0.012 | -0.64 | 6.05 |
|  |  | IL6 | 0.02 | 0.011 | 0.05 | 1.01 |
|  |  | PDL1 | 0.88 | 0.002 | 10.87 | 44.21 |
|  |  | TNFa | 1.03 | 1.000 | 10.21 | 8.98 |
| **6 months** | cDC2 | CD83 | 0.90 | 0.034 | 10.99 | 25.16 |
|  |  | IL10 | 0.87 | 0.417 | 0.39 | 1.00 |
|  |  | IL12 | 0.92 | 0.014 | 29.71 | 53.21 |
|  |  | IL6 | 0.95 | 0.068 | 53.67 | 73.46 |
|  |  | TNFa | 0.95 | 0.068 | 53.45 | 69.35 |
|  | cDCn | CD83 | 0.69 | 0.035 | 2.47 | 7.81 |
|  |  | IL10 | 0.82 | 0.822 | 0.10 | 0.12 |
|  |  | IL12 | 0.74 | 0.162 | 1.63 | 4.23 |
|  |  | IL1b | 0.87 | 0.162 | 0.61 | 4.07 |
|  |  | IL6 | 0.73 | 0.070 | 4.26 | 8.50 |
|  |  | IL8 | 1.12 | 0.056 | 12.02 | 6.78 |
|  |  | TNFa | 0.68 | 0.032 | 6.37 | 12.79 |
|  | Mono | CD83 | 0.95 | 0.043 | 16.35 | 40.16 |
|  |  | IL10 | 1.12 | 0.421 | -0.67 | -1.45 |
|  |  | IL12 | 0.97 | 0.148 | 21.05 | 33.82 |
|  |  | IL1b | 0.95 | 0.050 | -6.65 | 14.04 |
|  |  | IL27 | 0.93 | 0.022 | 45.80 | 60.38 |
|  |  | IL6 | 0.95 | 0.050 | 17.51 | 40.93 |
|  |  | IL8 | 0.96 | 0.056 | 9.90 | 25.66 |
|  |  | PDL1 | 0.91 | 0.014 | 40.10 | 78.55 |
|  |  | TNFa | 0.96 | 0.052 | 29.47 | 51.08 |
|  | pDC | CD83 | 0.95 | 0.043 | 16.35 | 40.16 |
|  |  | IL10 | 1.12 | 0.421 | -0.67 | -1.45 |
|  |  | IL12 | 0.97 | 0.148 | 21.05 | 33.82 |
|  |  | IL1b | 0.95 | 0.050 | -6.65 | 14.04 |
|  |  | IL27 | 0.93 | 0.022 | 45.80 | 60.38 |
|  |  | IL6 | 0.95 | 0.050 | 17.51 | 40.93 |
|  |  | IL8 | 0.96 | 0.056 | 9.90 | 25.66 |
|  |  | PDL1 | 0.91 | 0.014 | 40.10 | 78.55 |
|  |  | TNFa | 0.96 | 0.052 | 29.47 | 51.08 |

Cell subsets with no significant associations with PBMC viability.

**Table S4. Longitudinal changes in phenotypic functional characteristics in HEU**

| **Parameter** | **Cell subset** | **Marker** | **OR** | **FDR p** | **Birth** | **6 months** |
| --- | --- | --- | --- | --- | --- | --- |
| **Phenotype** | cDC2 | CD40 | 1.00 | 0.973 | 25.39 | 25.64 |
|  |  | CD83 | 0.99 | 0.931 | 54.98 | 57.16 |
|  |  | IL10 | 1.21 | 0.106 | 16.33 | 8.77 |
|  |  | IL12 | 0.97 | 0.441 | 31.09 | 47.46 |
|  |  | IL1b | 1.15 | 0.931 | 2.45 | 1.85 |
|  |  | IL27 | 0.98 | 0.594 | 26.81 | 42.38 |
|  |  | IL6 | 1.25 | 0.545 | 6.54 | 2.90 |
|  |  | IL8 | 0.98 | 0.594 | 37.07 | 54.37 |
|  |  | PDL1 | 0.98 | 0.594 | 54.59 | 67.07 |
|  |  | TNFa | 1.14 | 0.841 | 4.79 | 3.36 |
|  | cDCn | CD40 | 0.14 | 0.136 | 0.39 | 1.44 |
|  |  | CD83 | 1.04 | 0.783 | 13.88 | 12.16 |
|  |  | IL10 | 1.20 | 0.746 | 6.50 | 5.35 |
|  |  | IL12 | 0.72 | 0.184 | 3.31 | 8.35 |
|  |  | IL1b | 0.12 | 0.629 | 0.28 | 0.46 |
|  |  | IL27 | 0.53 | 0.136 | 1.38 | 4.15 |
|  |  | IL6 | 1.91 | 0.387 | 2.57 | 1.09 |
|  |  | IL8 | 0.75 | 0.135 | 3.13 | 7.38 |
|  |  | PDL1 | 0.73 | 0.136 | 3.11 | 5.77 |
|  |  | TNFa | 0.62 | 0.783 | 0.91 | 1.15 |
|  | Mono | CD40 | 1.10 | 1.000 | 2.09 | 1.80 |
|  |  | CD83 | 1.01 | 1.000 | 41.73 | 39.41 |
|  |  | IL10 | 1.02 | 1.000 | 29.65 | 24.43 |
|  |  | IL12 | 0.97 | 0.403 | 57.91 | 80.68 |
|  |  | IL1b | 0.98 | 1.000 | 16.10 | 24.50 |
|  |  | IL27 | 0.97 | 1.000 | 4.70 | 8.13 |
|  |  | IL6 | 1.17 | 0.373 | 9.14 | 3.41 |
|  |  | IL8 | 0.99 | 1.000 | 71.39 | 77.08 |
|  |  | PDL1 | 0.99 | 1.000 | 34.30 | 42.06 |
|  |  | TNFa | 0.98 | 1.000 | 19.49 | 27.46 |
|  | pDC | CD40 | 0.94 | 0.989 | 4.20 | 5.51 |
|  |  | CD83 | 0.97 | 1.000 | 32.80 | 38.43 |
|  |  | IL10 | 0.89 | 0.598 | 5.04 | 10.59 |
|  |  | IL12 | 0.98 | 1.000 | 5.73 | 6.91 |
|  |  | IL1b | 1.09 | 1.000 | 0.46 | 0.39 |
|  |  | IL27 | 1.00 | 0.989 | 14.93 | 15.12 |
|  |  | IL6 | 1.08 | 1.000 | 0.67 | 0.57 |
|  |  | IL8 | 0.97 | 0.485 | 34.90 | 45.92 |
|  |  | PDL1 | 0.95 | 0.294 | 21.15 | 35.99 |
|  |  | TNFa | 2.83 | 0.459 | 2.42 | 0.53 |
|  |  |  |  |  |  |  |

All markers.

**Table S5. Longitudinal changes in phenotypic functional characteristics in HUU**

| **Parameter** | **Cell subset** | **Marker** | **OR** | **FDR p** | **Birth** | **6 months** |
| --- | --- | --- | --- | --- | --- | --- |
| **Phenotype** | cDC2 | CD40 | 1.04 | 0.077 | 30.79 | 21.06 |
|  |  | CD83 | 1.02 | 1.000 | 63.47 | 59.76 |
|  |  | IL10 | 0.93 | 0.697 | 6.17 | 8.32 |
|  |  | IL12 | 0.99 | 1.000 | 44.47 | 45.99 |
|  |  | IL1b | 0.99 | 1.000 | 0.57 | 0.58 |
|  |  | IL27 | 1.06 | 0.010 | 70.60 | 55.30 |
|  |  | IL6 | 0.46 | 0.424 | 0.75 | 1.04 |
|  |  | IL8 | 1.02 | 0.178 | 62.19 | 55.86 |
|  |  | PDL1 | 1.17 | 0.004 | 92.30 | 78.70 |
|  |  | TNFa | 0.93 | 1.000 | 3.51 | 4.11 |
|  | cDCn | CD40 | 0.61 | 0.051 | 1.08 | 2.09 |
|  |  | CD83 | 0.65 | 0.001 | 5.53 | 12.85 |
|  |  | IL10 | 1.07 | 0.400 | 6.57 | 5.56 |
|  |  | IL12 | 0.66 | 0.001 | 3.31 | 11.43 |
|  |  | IL1b | 0.18 | 0.400 | 0.19 | 0.26 |
|  |  | IL27 | 0.75 | 0.014 | 3.78 | 7.14 |
|  |  | IL6 | 0.22 | 0.123 | 0.55 | 0.76 |
|  |  | IL8 | 0.82 | 0.014 | 4.18 | 8.34 |
|  |  | PDL1 | 0.66 | 0.004 | 3.78 | 8.52 |
|  |  | TNFa | 0.36 | 0.046 | 0.64 | 1.22 |
|  | Mono | CD40 | 1.82 | 0.060 | 2.54 | 1.04 |
|  |  | CD83 | 0.93 | 0.049 | 9.04 | 16.01 |
|  |  | IL10 | 0.96 | 0.387 | 13.50 | 17.28 |
|  |  | IL12 | 0.99 | 0.695 | 56.99 | 61.63 |
|  |  | IL1b | 1.03 | 0.827 | 2.66 | 2.55 |
|  |  | IL27 | 0.94 | 1.000 | 0.38 | 0.38 |
|  |  | IL6 | 0.66 | 0.012 | 2.16 | 4.87 |
|  |  | IL8 | 0.99 | 1.000 | 59.79 | 62.14 |
|  |  | PDL1 | 1.02 | 0.800 | 21.07 | 16.41 |
|  |  | TNFa | 0.89 | 0.312 | 4.60 | 6.57 |
|  | pDC | CD40 | 1.22 | 0.098 | 6.91 | 3.71 |
|  |  | CD83 | 0.93 | 0.031 | 24.05 | 34.61 |
|  |  | IL10 | 0.99 | 1.000 | 8.28 | 10.62 |
|  |  | IL12 | 0.70 | 0.031 | 1.82 | 4.50 |
|  |  | IL1b | 1.19 | 1.000 | 0.19 | 0.18 |
|  |  | IL27 | 1.05 | 0.197 | 20.65 | 16.13 |
|  |  | IL6 | 0.75 | 1.000 | 0.16 | 0.18 |
|  |  | IL8 | 0.96 | 0.121 | 25.13 | 34.06 |
|  |  | PDL1 | 1.03 | 0.267 | 31.98 | 28.19 |
|  |  | TNFa | 0.49 | 0.790 | 0.28 | 0.55 |
| **Function** | cDC2 | CD40 | 0.95 | 0.054 | 44.89 | 55.40 |
|  |  | CD83 | 1.00 | 0.961 | 24.99 | 25.16 |
|  |  | IL10 | 0.79 | 0.916 | 0.69 | 1.00 |
|  |  | IL12 | 0.96 | 0.388 | 47.68 | 53.21 |
|  |  | IL1b | 1.02 | 0.621 | 46.47 | 39.75 |
|  |  | IL27 | 0.96 | 0.054 | 14.09 | 21.94 |
|  |  | IL6 | 0.98 | 0.916 | 69.84 | 73.46 |
|  |  | IL8 | 1.04 | 0.054 | 20.48 | 9.18 |
|  |  | PDL1 | 0.77 | 0.003 | 2.15 | 10.19 |
|  |  | TNFa | 0.92 | 0.007 | 56.07 | 69.35 |
|  | cDCn | CD40 | 0.75 | 0.001 | 6.28 | 12.22 |
|  |  | CD83 | 0.89 | 0.234 | 6.27 | 7.81 |
|  |  | IL10 | 0.50 | 0.465 | 0.07 | 0.12 |
|  |  | IL12 | 0.75 | 0.026 | 1.56 | 4.23 |
|  |  | IL1b | 1.36 | 0.002 | 10.23 | 4.07 |
|  |  | IL27 | 1.25 | 0.002 | 17.83 | 9.79 |
|  |  | IL6 | 0.73 | 0.009 | 4.72 | 8.50 |
|  |  | IL8 | 1.25 | 0.002 | 15.82 | 6.78 |
|  |  | PDL1 | 1.13 | 0.002 | 30.86 | 20.51 |
|  |  | TNFa | 0.64 | 0.001 | 6.26 | 12.79 |
|  | Mono | CD40 | 0.88 | 0.0002 | 19.45 | 39.87 |
|  |  | CD83 | 1.07 | 0.005 | 57.43 | 40.16 |
|  |  | IL10 | 1.31 | 0.087 | -0.55 | -1.45 |
|  |  | IL12 | 1.15 | 0.001 | 57.57 | 33.82 |
|  |  | IL1b | 1.08 | 0.002 | 39.27 | 14.04 |
|  |  | IL27 | 0.95 | 0.134 | 53.27 | 60.38 |
|  |  | IL6 | 1.10 | 0.002 | 59.42 | 40.93 |
|  |  | IL8 | 1.03 | 0.136 | 35.02 | 25.66 |
|  |  | PDL1 | 0.99 | 0.576 | 76.10 | 78.55 |
|  |  | TNFa | 1.08 | 0.007 | 66.79 | 51.08 |
|  | pDC | CD40 | 0.77 | 0.001 | 16.23 | 32.74 |
|  |  | CD83 | 1.06 | 0.040 | 70.53 | 60.94 |
|  |  | IL10 | 5.72 | 0.208 | -0.01 | -0.12 |
|  |  | IL12 | 0.66 | 0.040 | -2.65 | 1.96 |
|  |  | IL1b | 1.08 | 0.230 | 6.05 | 4.72 |
|  |  | IL27 | 1.11 | 0.001 | 34.54 | 19.20 |
|  |  | IL6 | 0.36 | 0.040 | 1.01 | 1.66 |
|  |  | IL8 | 1.05 | 0.005 | 4.85 | -14.18 |
|  |  | PDL1 | 0.96 | 0.036 | 44.21 | 54.14 |
|  |  | TNFa | 0.80 | 0.0005 | 8.98 | 23.84 |

All markers.
